# Supplementary material for: A Multi-Epitope Recombinant Vaccine Candidate Against Bovine Alphaherpesvirus 1 and 5 Elicits Robust Immune Responses in Mice and Rabbits
Source: Vaccines (Basel). 2025 Oct 30;13(11):1115. doi: 10.3390/vaccines13111115 (PMC12656785; doi:10.3390/vaccines13111115)
Supplement: Supplementary file 1 [file vaccines-13-01115-s001.zip › vaccines-3877663_supplyment.pdf]

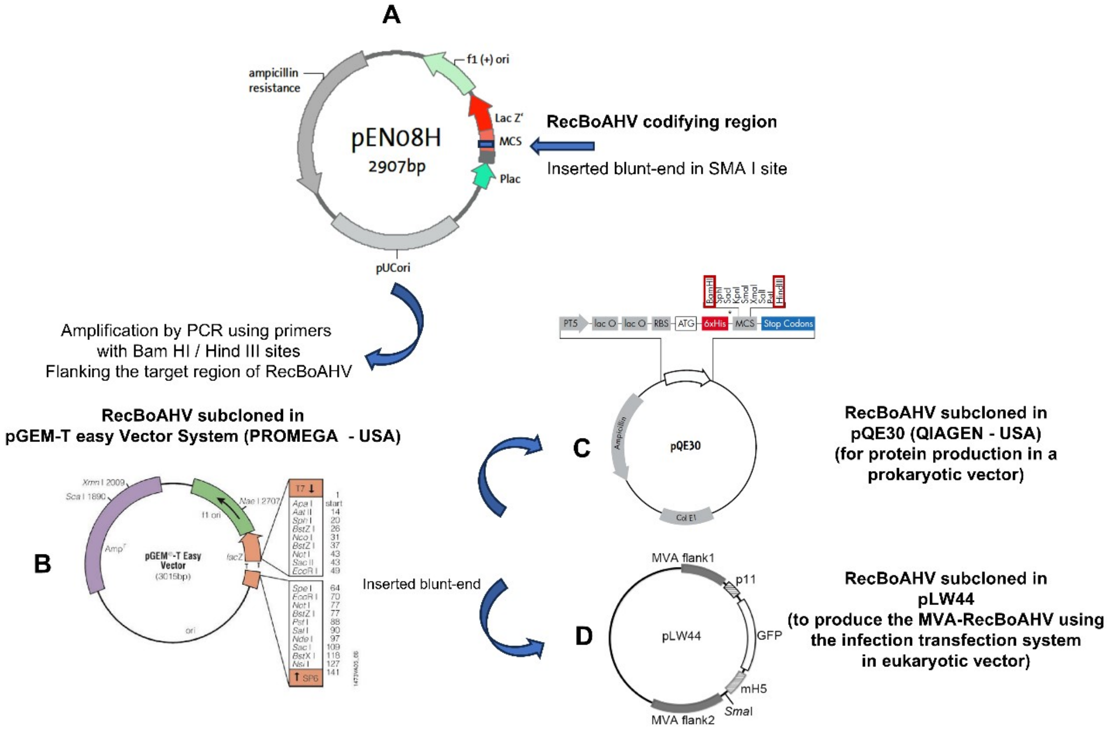

Figure S-1: Plasmid constructions with the RecBoAHV gene. (A) The synthetic gene encoding a recombinant BoAHV-1 and BoAHV-5 multi-epitope flexible protein (RecBoAHV) was custom-synthesized by Enthelecon (pEN08H, Germany). Primers for direct PCR amplification and insertion of BamHI and HindIII restriction enzyme sites to the 5′ and 3′ ends of the construct were synthesized by Integrated DNA Technologies (USA). (B) Purified PCR insert (gene coding the RecBoAHV) was subcloned into the pGEMT-easy vector system (Promega-USA). (C) The synthetic gene encoding the recombinant protein from plasmid pGEMT-easy vector and the expression plasmid pQE-30 (QIAGEN; USA) were restricted with BamHI and HindIII enzymes (PROMEGA – USA). The RecBoAHV coding gene was purified and subcloned into pQE-30 and used to transform the competent E. coli M15 strain for expression of the recombinant protein (RecBoAHV). (D) For the construction of the MVA-RecBoAHV Vector, the nucleotide sequence of the RecBoAHV multi-epitope protein cloned in vector pGEMT-easy was subcloned into pLW44 transfer plasmid (provided by Dr. Bernard Moss - NIH-NIAID) and confirmed by sequencing. The pLW44 transfer plasmid contains the green fluorescent protein coding sequence under the control of the mH5 early/late *Orthopoxvirus vaccinia* (VACV) promoter. MVA-RecBoAHV was produced using homologous recombination from infection (MVA) and transfection (pLW44-BoAHV) in a eucaryotic cell.

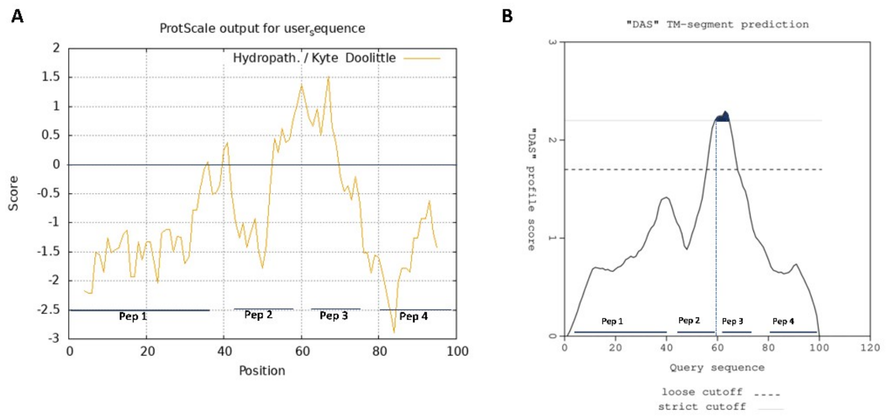

Figure S-2. Computation of hydropathicity and transmembrane prediction. (A) Kyte-Doolittle Hydropathy plot (http://web.expasy.org/protparam/). (B) Transmembrane segment prediction performed using DAS-Transmembrane Prediction server (tmdas.bioinfo.se). The position of the four peptides (Pep 1 to Pep 4) that compounds the multi-epitope RecBoAHV protein is pointed out in both graphics. The Kyte Doolittle transmembrane prediction has a cut-off above 1.6, showing in (A) the absence of a transmembrane segment. In DAS prediction (B) the strict cut off (2.2) showed a small portion of the transmembrane region initiating after the end of Pep 2 and finishing soon after the start of Pep 3.

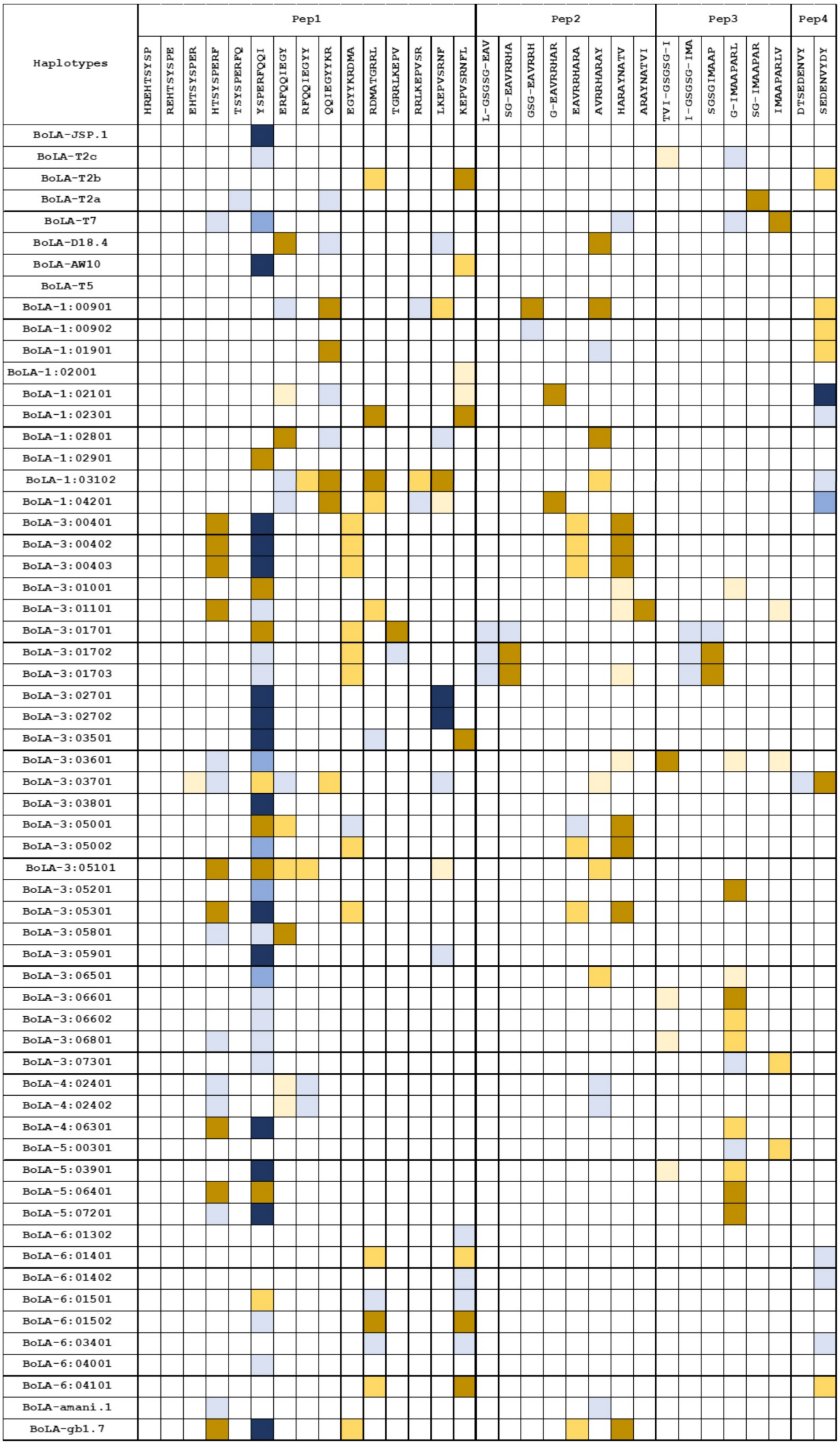

Weak Binders
>1.5
1.1-1.5
0.501-1

Strong Binders
0.5-0.1
0.09-0.05
<0.05

**Pep1**	HREHTSYSPERFQQIEGYYKRDMATGRRLKEPVSRNFL
**Pep2**	EAVRRHARAYNATVI
**Pep3**	IMAAPARLVEGQ
**Pep4**	DEDTSEDENVYDYIDGDSS

Figure S-3. Predicted binding affinity of BoLA class I haplotypes to RecBoAHV-derived peptides (Pep1 to Pep4) using the NetMHCpan tool (version 4.1). The heatmap represents the in-silico predicted binding strength of each peptide to a panel of bovine leukocyte antigen (BoLA) class I haplotypes. Peptides are grouped according to their position within the antigen: Pep1 (gB; B- and T-cell epitope), Pep2 (gD; B-cell epitope), Pep3 (gD; T-cell epitope), and Pep4 (tegument phosphoprotein; T-cell epitope). Color intensity indicates predicted binding affinity: darker shades correspond to stronger binding, while lighter shades indicate weak or non-binding interactions. Promiscuous epitopes are those showing consistently strong binding across multiple BoLA haplotypes, highlighting peptides with broad potential immunogenicity in diverse bovine populations.

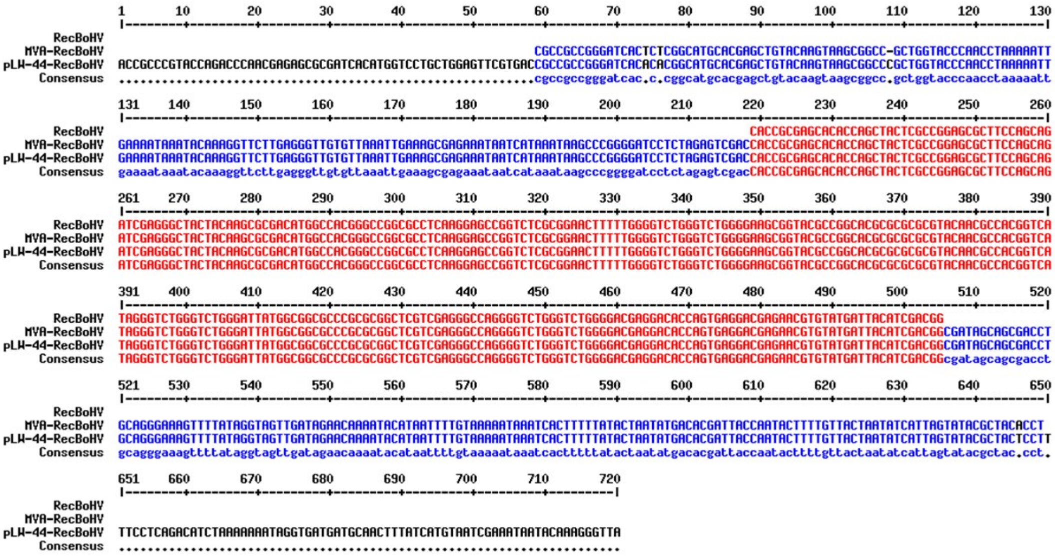

Figure S-4. Verification of the cloning of the target RecBoAHV sequence into the MVA-RecBoAHV genome by Sanger sequencing. A pair of primers annealing to the flanking regions of the pLW44 plasmid was used for PCR amplification. The obtained sequence was aligned with the RecBoAHV reference sequence and the pLW44 plasmid sequence to confirm correct insertion of the RecBoAHV gene into the MVA genome. This analysis ensures that the recombinant virus contains the intended antigenic insert and proper plasmid flanking regions.

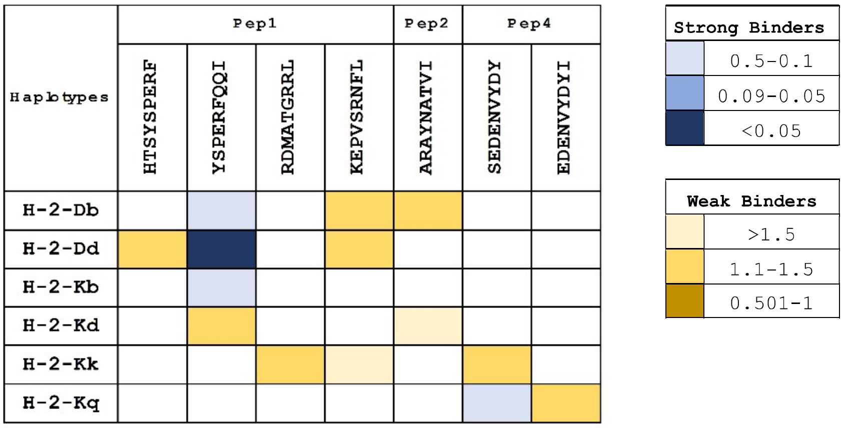

**Pep1**	HREHTSYSPERFQQIEGYYKRDMATGRRLKEPVSRNFL
**Pep2**	EAVRRHARAYNATVI
**Pep3**	IMAAPARLVEGQ
**Pep4**	DEDTSEDENVYDYIDGDSS

Figure S-5. Predicted binding affinity of MHC haplotypes to RecBoAHV-derived peptides (Pep1 to Pep4) using the NetMHCpan tool (version 4.1). The heatmap shows the in-silico predicted binding strength of each peptide to a panel of MHC haplotypes. Peptides are grouped according to their position within the antigen: Pep1 (gB; B- and T-cell epitope), Pep2 (gD; B-cell epitope), Pep3 (gD; T-cell epitope), and Pep4 (tegument phosphoprotein; T-cell epitope). Color intensity reflects predicted binding: darker shades represent strong binders, while lighter shades correspond to weak or non-binders. Promiscuous epitopes with broad MHC recognition are easily identifiable as consistently strong binding across multiple haplotypes, highlighting their potential for universal immunogenicity.

Glycoprotein B

Pep1-gB-RecBoAHV: HREHTSYSPERFQQIEGYYKRDMATGRRLKEPVSRNFL

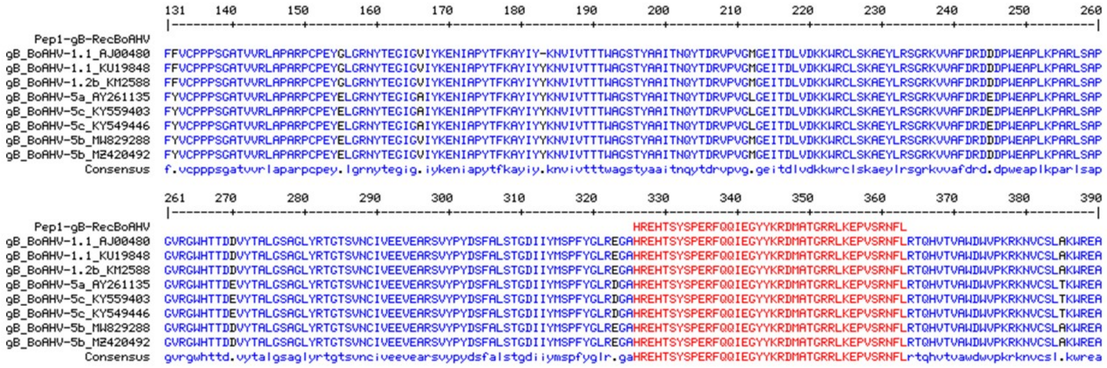

Glycoprotein D

Pep2-gD-RecBoAHV: EAVRRHARAYNATVI

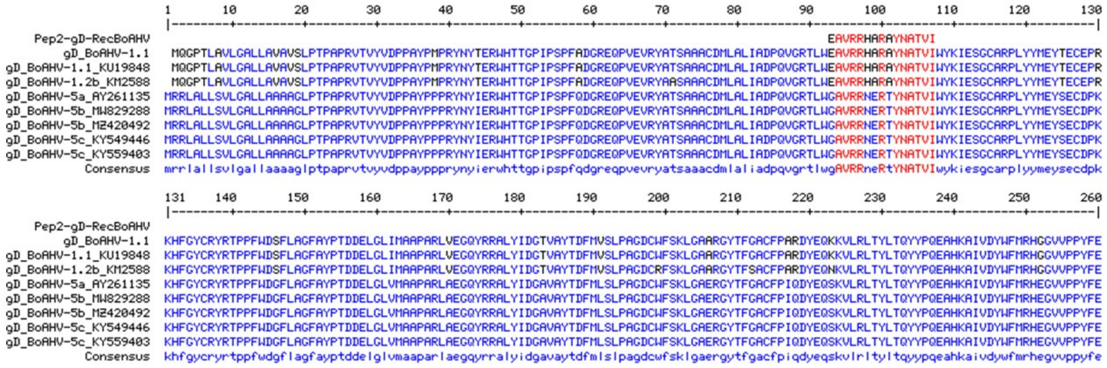

Glycoprotein D

Pep3-gD-RecBoAHV: IMAAPARLVEGQ

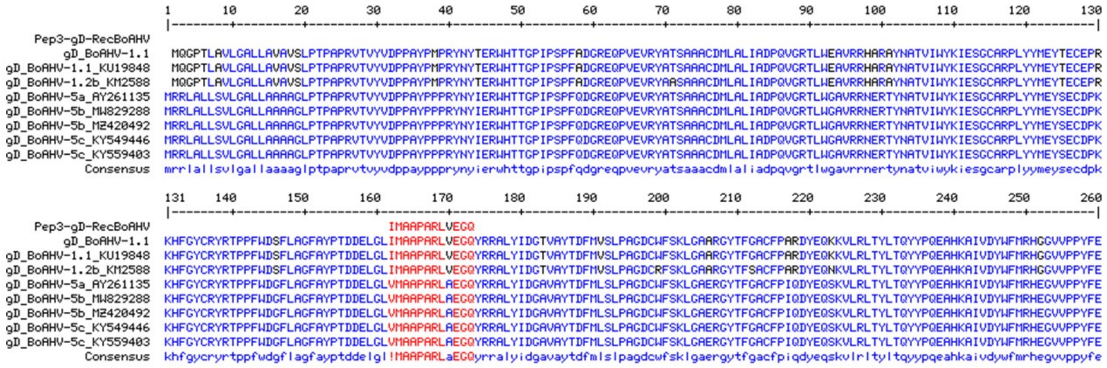

Tegument Phosphoprotein

Pep4-UL47_Tegument_RecBoAHV: DEDTSEDENVYDYIDGDSSD

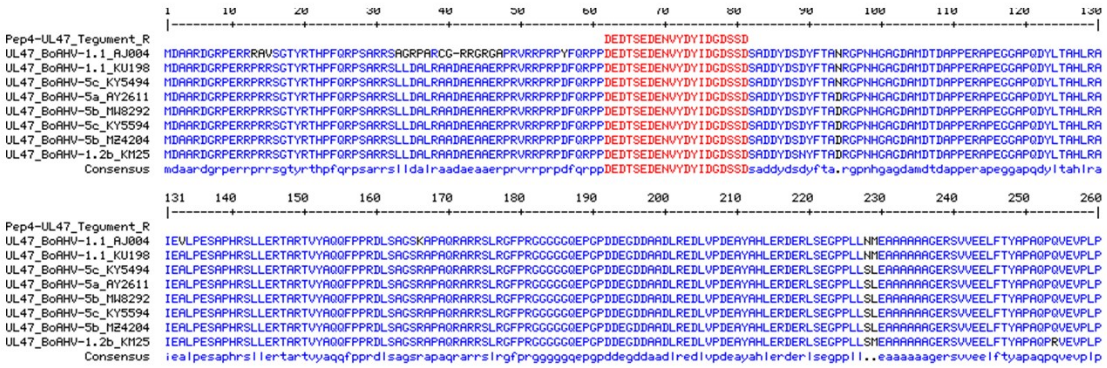

Figure S-6. Alignment of synthetic peptides Pep1 (gB; B- and T-cell epitope), Pep2 (gD; B-cell epitope), Pep3 (gD; T-cell epitope), and Pep4 (tegument phosphoprotein; T-cell epitope) with representative sequences of BoAHV-1.1 (AJ004801.1 – Switzerland; KU198480.2, strain Cooper – USA), BoAHV-1.2 (KM258880.1, strain K22 – USA), and BoAHV-5 (5a: Y2611359.1, strain SV507.99 – Brazil; 5b: MW829288, strain A663 – Brazil, and MZ420492, strain 674/10 – Argentina; 5c: KY549446.1, strain ISO 97/45 – Brazil, and KY559403.2, strain P160/96 – Brazil). The alignment revealed complete identity (100%) for Pep1 across all sequences. For Pep2, 100% identity was observed with BoAHV-1.1 and BoAHV-1.2 (AJ004801.1, KU198480.2, and KM258880.1), while showed 73% identity for BoAHV-5 (Y2611359.1-5a, MW829288-5b, MZ420492-5b, KY549446.1-5c, and KY559403.2-5c). For Pep3, 100% identity was observed with BoAHV-1.1 and BoAHV-1.2 (AJ004801.1, KU198480.2, and KM258880.1), whereas 93% identity was observed with BoAHV-5 (Y2611359.1-5a, MW829288-5b, MZ420492-5b, KY549446.1-5c, and KY559403.2-5c). Pep4 exhibited 100% identity with all sequences analyzed.
